# Long, Uniform Lactobacilli (Döderlein's Bacteria): A New Risk Factor for Postoperative Infection After First-Trimester Abortion

**DOI:** 10.1155/S106474499500041X

**Published:** 1995

**Authors:** Jens-Jörgen Platz-Christensen, Carl Påhlson, Per-Göran Larsson

**Affiliations:** 1 Department of Obstetrics and Gynaecology University of Gothenburg Gothenburg Sweden; 2 Dept. of Obstetrics and Gynaecology Central Hospital Skövde S-54185 Sweden; 3 Department of Infectious Diseases and Clinical Microbiology University of Uppsala Uppsala Sweden

## Abstract

*Objective:* The production of hydrogen peroxide (H_2_O_2_) from different strains of lactobacilli in the vagina has been proposed to play one of the most important protective roles in the vaginal defense system. New data have, however, suggested that Döderlein's bacteria, with the morphological appearance of long lactobacilli, have a low production of H_2_O_2_ . The purpose of the present study was to correlate the morphology of lactobacilli with the incidence of infection following legal abortion.

*Methods:* Seven hundred sixty-nine women with lactobacilli but without *Chlamydia trachomatis* or bacterial vaginosis in their vaginal wet smears who were to undergo legal abortions were divided into 6 different groups according to the morphological appearance of the lactobacilli. The postoperative infection rates in these different groups were compared. A phenotypic classification of some of the lactobacilli was performed.

*Results:* The presence of Döderlein's bacteria compared with a mixed flora of lactobacilli increased the risk of postoperative infection 3 times [relative risk (RR) = 3.0; 95% confidence interval (CI), 1.5-6.3]. After a logistic regression analysis, the only independent risk factors were the presence of Döderlein's bacteria and earlier gestational age.

*Conclusions:* We found that the lactobacilli regarded as commensal organisms and “normal, healthy lactobacilli” in the vagina were present in only 18% of these women and that their presence might be hazardous. Therefore, we must reconsider our concept of the “normal” lactobacilli in the vaginal wet smears of healthy women.


Infectious Diseases in Obstetrics and Gynecology 3:102–109 (1995)
© 1995 Wiley-Liss, Inc.

### Long, Uniform Lactobacilli (Döderlein's Bacteria): A New Risk Factor for Postoperative Infection After First-Trimester Abortion

**Jens-Jörgen Platz-Christensen, Carl Påhlson, and Per-Göran Larsson**

*Department of Obstetrics and Gynaecology, University of Gothenburg, Gothenburg (J.-J.P.-C.), Central Hospital, Skövde (P.-G.L.), and Department of Infectious Diseases and Clinical Microbiology, University of Uppsala, Uppsala (C.P.), Sweden*

### Abstract

***Objective:*** The production of hydrogen peroxide ($H_2O_2$) from different strains of lactobacilli in the vagina has been proposed to play one of the most important protective roles in the vaginal defense system. New data have, however, suggested that Döderlein's bacteria, with the morphological appearance of long lactobacilli, have a low production of $H_2O_2$. The purpose of the present study was to correlate the morphology of lactobacilli with the incidence of infection following legal abortion.

***Methods:*** Seven hundred sixty-nine women with lactobacilli but without *Chlamydia trachomatis* or bacterial vaginosis in their vaginal wet smears who were to undergo legal abortions were divided into 6 different groups according to the morphological appearance of the lactobacilli. The postoperative infection rates in these different groups were compared. A phenotypic classification of some of the lactobacilli was performed.

***Results:*** The presence of Döderlein's bacteria compared with a mixed flora of lactobacilli increased the risk of postoperative infection 3 times [relative risk (RR) = 3.0; 95% confidence interval (CI), 1.5–6.3]. After a logistic regression analysis, the only independent risk factors were the presence of Döderlein's bacteria and earlier gestational age.

***Conclusions:*** We found that the lactobacilli regarded as commensal organisms and "normal, healthy lactobacilli" in the vagina were present in only 18% of these women and that their presence might be hazardous. Therefore, we must reconsider our concept of the "normal" lactobacilli in the vaginal wet smears of healthy women. © 1995 Wiley-Liss, Inc.

Key words

Bacterial vaginosis, endometritis, pelvic inflammatory diseases, legal abortion

The reported incidence of postoperative infection after first-trimester abortion is 4–12%.[1–3] Many attempts have been made to identify risk groups. The patients considered to be at risk are women with histories of pelvic inflammatory disease (PID),[4] women with cervical infection with either *Chlamydia trachomatis* or *Neisseria gonorrhoea*,[5] and women with bacterial vaginosis.[6] Other risk groups are women who have had at least 1 legal abortion,[4] nulliparous women,[7] women at later gestational ages,[8] women with vaginal leucocytosis,[9] and women whose abortions are performed bv less-experienced surgeons.[8]

A recently published study reported that the preoperative treatment of bacterial vaginosis with metronidazole before legal abortion reduces the incidence of postoperative infection from 12.2 to 3.8%.[10] Nevertheless, women with lactobacilli in

Address correspondence/reprint requests to Dr. Per-Göran Larsson, Dept. of Obstetrics and Gynaecology, Central Hospital, S-541 85 Skövde, Sweden.

Clinical Study

Received January 26, 1995

Accepted July 17, 1995

**TABLE 1. Morphological appearances of lactobacilli seen in vaginal wet smears and their frequency in women without *C. trachomatis* infection or bacterial vaginosis presenting for abortions**

| Morphological type of lactobacilli | Characteristic | Size (μm) | Frequency % | No. |
|---|---|---|---|---|
| 1. Variable, straight scanty | Pleomorph | 2–8 | 48.5 | 373 |
| 2. Uniform, long (Döderlein's bacteria) | Uniform | 4–6 | 17.5 | 135 |
| 3. Uniform, short | Uniform | 2–4 | 21.1 | 162 |
| 4. Fusoform | Uniform | 8–20 | 6.9 | 53 |
| 5. Curved | Uniform | 3–6 | 4.2 | 32 |
| 6. Mixed | Pleomorph | | 1.8 | 14 |
| Total | | | 100.0 | 769 |

the vagina who have neither bacterial vaginosis nor *C. trachomatis* still develop postoperative infections.[6]

In the first microbiological study of the human vaginal flora in 1892, Albert Döderlein[11] described the long, straight, Gram-positive bacteria as the normally predominating species. These bacteria were subsequently classified under the genus *Lactobacillus*. Döderlein's observation has been so generally accepted that these bacteria are still called Döderlein's bacilli. However, within the genus *Lactobacillus*, the morphology can vary from long, slender rods to coryneform coccobacilli. Some species are uniform in size, while others vary from 0.5 to 6.0 μm.[12] The genus *Lactobacillus* consists of at least 71 different species. Microbiological studies have shown that the lactobacilli occurring in the vagina represent a great number of species and strains, the most frequent being *Lactobacillus acidophilus*.[13] Characteristic of Döderlein's bacteria, according to photographs in Döderlein's original article,[11] is their uniform length, usually between 5.0 and 6.0 μm. These uniform and long gram-positive rods are the type illustrated in all textbooks as the normal lactobacilli in the vaginal flora. The most common lactobacilli seen in wet smears, however, have a pleomorphological appearance that differs from the Döderlein's type.[14] Lactobacilli have the ability to produce a variety of bactericidal substances such as inhibitory proteins,[15] lactic acid,[16] "lactocidins,"[17] and $H_2O_2$.[18] It has been suggested that some lactobacilli strains normally found in the vagina have different protective capabilities against infections such as bacterial vaginosis[14,19] and *N. gonorrhoea*,[20] depending on their production of these different antibacterial substances. Eschenbach et al.[19] have shown that the lactobacilli recovered from women with bacterial vaginosis have a lower ability to produce $H_2O_2$ compared with the lactobacilli isolated from healthy women.[11]

The purpose of the present study was to correlate morphologically different lactobacilli by wet smear microscopy with the risk for postoperative infection after first-trimester abortion in women not having bacterial vaginosis, chlamydia, gonorrhea, or trichomoniasis.

### MATERIALS AND METHODS

Patients with bacterial vaginosis (defined according to the criteria of Amsel et al.),[21] trichomoniasis, or *C. trachomatis* infection were not included in the study. *C. trachomatis* was cultured in Gothenburg and diagnosed in Skövde using a combination of enzyme-linked immunosorbent assay (ELISA) (Chlamydiazyme) and indirect fluorescence (FA) (Microtrack). Culturing for *N. gonorrhoeae* was done in Gothenburg. All cultures for *N. gonorrhoeae* were negative; because of the low gonorrhea incidence in Sweden,[22] culturing was not done in Skövde. To ascertain if the diagnoses were uniform, we evaluated all specimens on air-dried smears in accordance with our previously published method.[23]

### Lactobacilli Morphotype

The lactobacilli were divided into the following 6 groups (Table 1) according to the predominating morphology (Fig. 1):

1. *Variable, straight, scanty lactobacilli:* Bacterial size of 2–8 μm. A scanty flora with a high degree of size variation. (This pleomorphic appearance was also seen after pure culturing.)

2. *Uniform, long lactobacilli:* Bacterial size of

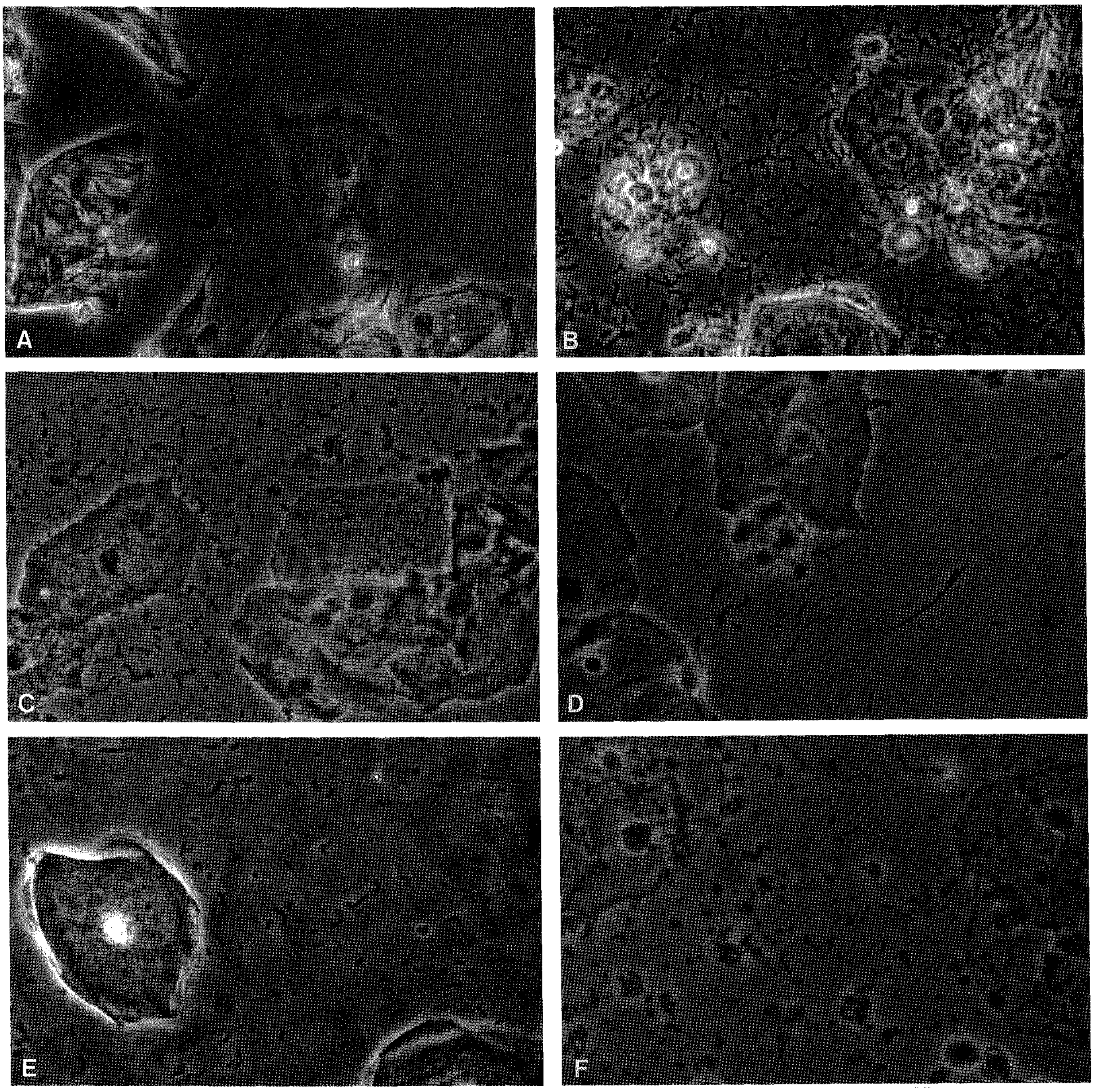

**Fig. 1.** Lactobacilli divided according to morphological appearances in wet smears. **A:** variable, straight, scanty lactobacilli. **B:** uniform, long lactobacilli. **C:** uniform, short lactobacilli. **D:** very long, fusoform lactobacilli. **E:** curved lactobacilli. **F:** mixed form.

4–6 μm. (These uniform lactobacilli, corresponding to Döderlein's bacilli, were often seen in high numbers.)

3. *Uniform, short lactobacilli:* Bacterial size of 2–4 μm. Similar appearance to uniform, long lactobacilli but short.

4. *Very long, fusoform lactobacilli:* Bacterial size of 8–20 μm. A striking feature is that they can be almost as long as a spermatozoa. (This bacterium is termed *Leptothrix* by some authors).[24]

5. *Curved lactobacilli:* Bacterial size of 3–6 μm. Mostly occurring as a scanty flora of lactobacilli. (In stained smears, they can be mistaken for "curved rods" or *Mobiluncus*, but differ in that their ends are not crescent-shaped and they are much thicker and more bent.)

6. *Mixed form:* Lactobacilli not classified into one of the other groups.

For our calculations, we divided the lactobacilli into 3 groups: 1) variable, straight, scanty; 2) uniform, long (Döderlein); and 3) other. A single-blind evaluation of the air-dried, rehydrated wet smears was carried out after the study was completed.

### Lactobacilli Culture

The lactobacilli from 63 patients were cultured at the Department of Clinical Microbiology, University of Uppsala, Sweden. The determination of species was accomplished by phenotypic analyses based on the fermentation pattern of 18 different carbohydrates in accordance with the Virginia Polytechnical Institute manual.[25] This process was combined with enzyme assays of $H_2O_2$ production according to Eschenbach et al.[19] The morphological appearance after culturing was studied using Gram's stain as well as the ability to grow under aerobic and anaerobic conditions.

### Operation

A vacuum aspiration was performed on each patient within 1 week of her outpatient visit and before the 12th completed week of gestation. Preoperative ripening of the cerivx was performed in nulliparous women using intravaginally administered prostaglandin [gemeprost (Cervagem)] in Skövde or an intracervical tent (Dilapan hydroscopic cervical dilator, Gynotech, Inc., USA) in Göteborg. Each patient's medical record was evaluated at a follow-up visit 1 month later. The diagnosis of postoperative infection after the abortion was made if tenderness over the uterus or adnexa was palpated and at least 1 of the following 5 criteria was noted within 28 days of the aspiration: 1) abnormal or purulent vaginal discharge after the first postoperative week, 2) abnormal bleeding after the third postoperative day, 3) temperature >38°C for longer than 24 h, 4) palpable adnexal mass, or 5) erythrocyte sedimentation rate >30 mm.

The statistical technique used was logistic regression analysis with maximum probability estimates,[26] chi-square, and relative risks (RRs). $P$ values >0.05 were considered not significant (NS).

The study was approved by the regional ethics committee.

### RESULTS

During the study period, 1,224 women visited our outpatient clinics for first-trimester legal abortions. After the exclusion of women with bacterial vaginosis, chlamydia, gonorrhea, or trichomoniasis, 889 women remained. Inappropriate material, i.e., interfering blood, which made it impossible to make a correct diagnosis, excluded 33 patients. Also excluded were 7 women with inadequately emptied uterine cavities. In addition, the wet smears from 80 patients were lost. In all, we were able to classify the lactobacilli in 769 women. Variable, straight, scanty lactobacilli were found in 373 (48%); long, uniform lactobacilli (Döderlein's bacteria) in 135 (18%); short lactobacilli in 162 (21%); fusoform lactobacilli in 53 (7%); curved lactobacilli in 32 (4%); and mixed lactobacilli in 14 (2%). For our calculations, the short, fusoform, curved, and mixed forms of lactobacilli were grouped under "other forms of lactobacilli." This group consisted of 261 (34%) women (Table 1).

The demographic data showed a mean age of 25.9 years. Of the study population, 29% of the women had histories of legal abortion and 61% of the women were nulliparous. After the abortion, an intrauterine device (IUD) was inserted in 176 (23%) women. For cervical ripening, intravaginal prostaglandin was used in 101 (13%) women and an intracervical tent was used in 259 (34%) women.

During the study, 28 cases of postoperative PID occurred. In addition to tenderness palpated over the uterus or adnexa, 19 (67%) of these 28 women had fever, 2 (7%) had abnormal bleeding, 6 (21%) had abnormal discharges, and 1 (3.5%) had an erythrocyte sedimentation rate of >30 mm.

The average time between abortion and postoperative infection was 4 days (range 1–23). The infection rates in the 2 hospitals were 3.0% and 4.4% (NS), respectively. The relationship of different factors with postoperative infection is shown in Table 2. Among the 373 women, there were 11 (8.1%) cases of postoperative infection in women with Döderlein's bacteria compared with 11 (2.9%) cases in women with variable, straight, scanty lactobacilli and 6 (2.7%) cases in women with other types of lactobacilli ($P = 0.008$). The RR of developing a postoperative infection in the women having Döderlein's bacteria was 3.0 (95% CI, 1.5–6.3) compared with women having other morphological types of lactobacilli.

TABLE 2. Post-abortion PID associated with various factors of different lactobacilli seen in wet smears of women without *C. trachomatis* infection or bacterial vaginosis

| Clinical sign | No. (%) | Normal | No. (%) | Missing data | *P* value | RR | 95% CI |
|---|---|---|---|---|---|---|---|
| Döderlein, uniform long | 11/135 (8.1) | Other lactobacilli | 17/634 (2.7) | 0 | <0.01 | 3.0 | 1.5–6.3 |
| Döderlein, uniform long | 11/135 (8.1) | Variable, straight | 11/373 (2.9) | 0 | 0.011 | 2.8 | 1.2–6.2 |
| Uniform long & short | 16/297 (5.4) | Variable, straight | 11/373 (2.9) | 0 | NS | — | |
| Leukocytosis | 14/225 (6.2) | No leukocytosis | 14/536 (2.6) | 8 | <0.05 | 2.4 | 1.2–4.9 |
| IUD inserted | 7/176 (4.0) | No IUD | 21/585 (3.6) | 8 | NS | — | |
| Cervical ripening | 10/360 (2.8) | No ripening | 18/401 (4.5) | 8 | NS | — | |
| Previous legal abortion | 10/224 (4.5) | No abortion | 18/536 (3.4) | 9 | NS | — | |
| Nulliparious | 17/474 (3.6) | Parous | 11/286 (3.8) | 9 | NS | — | |

Vaginal leucocytosis appeared to be a risk factor for postoperative infection in the first statistical analysis with an RR of 2.4 and a 95% CI of 1.2–4.9. Logistic regression analysis with maximum probability estimates done with the Statistical Analysis System program revealed only 2 explanatory variables: Döderlein's bacteria and earlier gestational age. Vaginal leukocytosis, insertion of an IUD in connection with the abortion, cervical ripening, previous legal abortion, parity, and the operating physician's experience were not found to be explanatory variables.

The infection rate in relation to gestational age showed a linear decline from the 7th week (11.0%) until the 11th week (1.6%). Neither the 6th nor the 12th week was included in the logistic analysis because of small numbers (Fig. 2).

The medical experience of the operating physician was considered because it has been reported to be of importance.[8] Senior attending surgeons, attending surgeons, or resident surgeons under training supervised by attending surgeons performed the abortions. As many as 35 different surgeons took part in the study. As described earlier, the senior attending surgeons, with more operative experience, showed the highest infection rate (5.6% compared with 3.3% for the attending surgeons), but this rate did not differ significantly from those of the other surgeons (NS) (Table 3). No correlation was found between the number of abortions performed by each surgeon and the infeciton rate.

The microbiological classification of the lactobacilli showed a great variety of species (Table 4). The most common was a genus classification to *Lactobacillus* spp. due to irreproducible fermentation patterns. $H_2O_2$ production was less pronounced in the group of women with uniform,

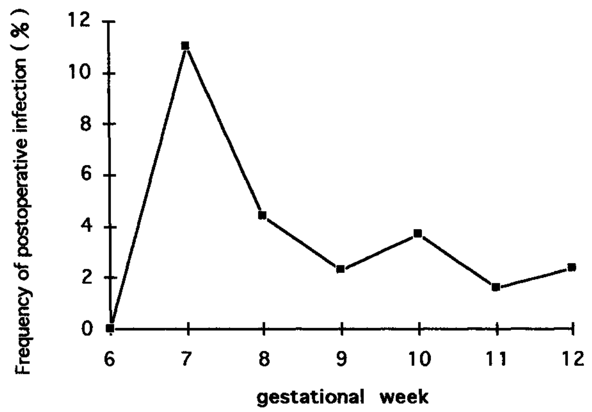

Fig. 2. Frequency of postoperative infections (%) after abortions in relation to gestational weeks. There is a statistical decline between the 7th and 11th week but the differences are small.

long lactobacilli (Döderlein's bacteria) compared with the women with other morphological groups.

### DISCUSSION

In an attempt to reduce the risk of postoperative infection associated with first-trimester abortion, several investigators have studied the criteria for prophylactic treatment with antibiotics. The risk groups identified so far have been women with positive cultures of *N. gonorrhoeae* or *C. trachomatis*[5] and women with bacterial vaginosis.[10] Since the incidence of *N. gonorrhoeae* in Scandinavian countries is low,[22] screening is often performed only for *C. trachomatis* infection in women presenting for first-trimester abortions.[5]

Many post-abortion infections occur within 1 week following surgery. The question has been raised as to whether or not these early infections are caused primarily by *C. trachomatis*.[10] Early infec-

**TABLE 3. Surgeon's medical training and postoperative infection rate in women without *C. trachomatis* infection or bacterial vaginosis**

| Surgeon | No. of patients | Infection No. | Infection % | Range |
|---|---|---|---|---|
| Resident surgeons (10) | 166 | 5 | 3.0 | 0.0–4.8 |
| Attending surgeons (14) | 459 | 15 | 3.3 | 0.0–12.5 |
| Senior attending surgeons (11) | 144 | 8 | 5.6 | 0.0–11.1 |
| All surgeons (35) | 769 | 28 | 3.7 | |

**TABLE 4. $H_2O_2$ production in relation to morphological type and phenotype of lactobacilli in women without *C. trachomatis* infection or bacterial vaginosis presenting for abortions**

| Morphological type | $H_2O_2$ production (%) | Phenotyped lactobacilli |
|---|---|---|
| Variable, straight | 74 | *Lactobacillus* spp.<br>*L. acidophilus, L. fermentum* |
| Uniform, long Döderlein's bacteria | 25 | *Lactobacillus* spp.<br>*L. acidophilus, L. crispatus* |
| Uniform, short | 50 | *Lactobacillus* spp.<br>*L. casei, L. plantarum* |
| Fusoform | 70 | *L. gasseri* |
| Curved | | *Lactobacillus* spp. |

tions occurring mostly in the endometrial cavity may not be as hazardous to future fertility as salpingitis developing 2–4 weeks after surgery.[27] This type of salpingitis is probably more often caused by *C. trachomatis*.[2] Studies published on the role of *C. trachomatis* in postoperative infection after abortion, however, do not divide the infections into early and late.[1–3,5] Nevertheless, early infections still cause a great deal of discomfort and morbidity as well as longer hospitalizations for patients.

A previously suggested risk factor, later gestational age,[4] was not confirmed. The highest infection rate occurred during the 7th week, although the differences were small. In the future, antiprogesterone (mifepristone) and prostaglandin will be used to terminate pregnancies at earlier gestational ages. Nevertheless, the results of our study are significant for the majority of women who will still have to undergo surgical abortions.

Although previously recognized as a risk factor, the experience of the surgeon was not shown to be important in our study.

In a clinical study such as ours, establishing strict criteria for the diagnosis of post-abortion infection is difficult. The use of laparoscopy for the diagnosis of postoperative infection after first-trimester abortion has not been proved beneficial. We made the diagnosis of postoperative infection based on palpation of tenderness over the uterus or adnexa plus at least 1 of 5 previously mentioned clinical criteria. We did not find in a study of the literature such a strict definition used in other studies of post-abortion infection.[1–10,28,29]

During abortion, microorganisms could conceivably ascend to the endometrial cavity[28] and cause infection in some patients. This route might explain infection in a patient with bacterial vaginosis, a condition characterized by a high number of bacteria/ml of vaginal secretion. Analogously, women with Döderlein's bacteria often have high numbers of bacteria, which might explain their higher infection rate. However, other factors such as weaker defense mechanisms due to low $H_2O_2$ production might also be responsible.

The isolation and classification of different *Lactobacillus* spp. using phenotypic characters are difficult and time-consuming and, therefore, not recommended.[30] In our study, the lactobacilli were phenotypically characterized most commonly as *Lactobacillus* spp., a result confirmed by other investigators.[29,30] Genetic analyses by deoxy-sequencing of the 16 s rRNA gene and alignment analysis of the Gene-bank database have shown that further microbiological research needs to be done before culturing can be used to identify these different lactobacilli. The *Lactobacillus* spp. cultured may or may not be the same *Lactobacillus* classified by microscopy. However, there are difficulties in culturing lactobacilli. In some patients with variable, scanty lactobacilli in their wet smears, the cultures are negative for lactobacilli. In addition, changing the growth characteristics produces different fermentation patterns. Whether or not morphological changes appear among the same *Lactobacillus* spp. in vivo during the normal menstrual cycle is unknown. The classification we have presented is based on only one smear taken on one occasion. Until progress is made, physicians may have to rely on microscopy to determine which women are at a greater risk of postoperative infection after an abortion. No prospective study has been done regarding the preoperative treatment of women with Döderlein's bacteria undergoing legal abortions and this bacteria's effect on the postoperative infection rate. Regardless of what kind of *Lactobacillus* spp. the phenotypic classification results show, we have found a simple and useful instrument for the morphological classification of lactobacilli. Lactobacilli presenting as the "normal, healthy lactobacilli" of the vaginal flora were present in only 18% of the women in our study. Furthermore, these lactobacilli might be hazardous for women. The most common type of lactobacilli, which nearly half of our patients harbored, was the variable, straight, scanty kind. Perhaps we should consider this type to be the normal lactobacilli of the vagina.

The new form of vaginal discharge described by Horowitz et al.[24] as vaginal lactobacillosis caused by long, anaerobic lactobacilli is probably consistent with our type 4, fusoform lactobacilli, genotyped to *Lactobacillus gasseri*. This type of lactobacilli, in our study, did not appear to be virulent as it did not correlate with postoperative infection.

Our study points out the need to reconsider our concept of "normal" lactobacilli and to give priority to research on human lactobacilli.

### ACKNOWLEDGMENTS

We express our sincere appreciation to Anita Johnson, PhD, for valuable assistance with the statistical analysis. Financial support was provided by grants from the Skaraborg County Council Research Committee and the Gothenburg Medical Society.

### REFERENCES

1. Möller BR, Ahrons S, Laurin J, Mårdh P-A: Pelvic infection after elective abortion associated with *Chlamydia trachomatis*. Obstet Gynecol 59:210–213, 1982.
2. Osser S, Persson K: Postabortal pelvic infection associated with *Chlamydia trachomatis* and the influence of humoral immunity. Am J Obstet Gynecol 150:699–703, 1984.
3. Qvigstad E, Skaug K, Jerve F, Fylling P, Ulstrup JC: Pelvic inflammatory disease associated with *Chlamydia trachomatis* infection after therapeutic abortion. Br J Vener Dis 59:189–192, 1983.
4. Heisterberg L, Sonne-Holm S, Andersen JT, Hebjörn S, Dyring-Andersen K, Hejl BL: Risk factors in first-trimester abortion. Acta Obstet Gynecol Scand 61:357–360, 1982.
5. Skjeldestad FE, Tuveng J, Solberg AG, Molne K, Dalen A, Buhaug H: Induced abortion: *Chlamydia trachomatis* and postabortal complications. A cost-benefit analysis. Acta Obstet Gynaecol Scand 67:525–529, 1988.
6. Larsson P-G, Bergman B, Forsum U, Platz-Christensen JJ, Påhlson C: *Mobiluncus* and clue cells as predictors for PID after first trimester abortion. Acta Obstet Gynecol Scand 68:217–220, 1989.
7. Bokström H, Wiqvist N: Preoperative dilatation of the cervix at legal abortion with a synthetic, fast-swelling hygroscopic tent. Acta Obstet Gynaecol Scand 68:313–318, 1989.
8. Moberg P, Sjöberg B, Wiqvist N: The hazards of vacuum aspiration in late first trimester abortions. Acta Obstet Gynecol Scand 154:113–118, 1975.
9. Hamark B, Forssman L: Postabortal endometritis in chlamydia-negative women: Association with preoperative clinical signs of infection. Gynecol Obstet Invest 31:102–105, 1991.
10. Larsson P-G, Platz-Christensen JJ, Thejls H, Forsum U, Påhlson C: Incidence of pelvic inflammatory disease after first-trimester legal abortion in women with bacterial vaginosis after treatment with metronidzaole: A double-blind, randomized study. Am J Obstet Gynecol 166: 100–103, 1992.
11. Döderlein A: Das Scheidensekret und seine Bedeutung für das Puerperalfieber. Leipzig, Germany: Verlag von Eduard Besold, 1982.
12. Kandler O, Weiss N: Genus *Lactobacillus*. In Sneath PHA, Mair NS, Sharpe ME, Holt JG (eds): Bergery's Manual of Systematic Bacteriology. Volume 2. Baltimore: Williams & Wilkins, pp 1209–1211, 1986.
13. Rogosa M, Shape ME: Species differentiation of human vaginal lactobacilli. J Gen Microbiol 23:197–201, 1960.
14. Påhlson C, Larsson P-G: The ecologically wrong vaginal lactobacilli. Med Hypotheses 36:126–130, 1991.
15. Mehta AM, Patel KA, Dave PJ: Isolation and purification of an inhibitory protein from *Lactobacillus acidophilus* AC1. Microbios 37:37–43, 1983.
16. Tramer J: Inhibitory effect of *Lactobacillus acidophilus*. Nature 211:204–205, 1966.
17. Vincent JG, Veomett RC, Riley RF: Antibacterial activity associated with *Lactobacillus acidophilus*. J Bacteriol 78: 477–484, 1959.
18. Wheter DM, Hirsch A, Mattrick ATR: Possible identity of "lactobacillin" with hydrogen peroxide produced by lactobacilli. Nature 170:623–624, 1952.
19. Eschenbach D, Davick PR, Williams BL, Klebanoff SJ, Young-Smith K, Critchlow CM, Holmes KK: Prevalence of hydrogen peroxide-producing *Lactobacillus* species in normal women and women with bacterial vaginosis. J Clin Microbiol 27:251–256, 1989.
20. Zheng H-y, Alcorn TM, Cohen MS: Effects of $H_2O_2$-

producing lactobacilli on *Neisseria gonorrhoeae* growth and catalase activity. J Infect Dis 170:1209–1215, 1994.

21. Amsel R, Totten PA, Spiegel CA, Chen K, Eschenbach DA, Holmes KK: Nonspecific vaginitis. Diagnostic criteria and microbial and epidemiologic associations. Am J Med 74:14–22, 1983.
22. Department of Epidemiology, Stockholm, Sweden: The Yearly Incidence of Gonorrhoea in Sweden 1912–1989. Stockholm: National Bacteriological Laboratory, 1990.
23. Larsson P-G, Platz-Christensen JJ: Enumeration of clue cells in rehydrated air-dried vaginal wet smears for the diagnosis of bacterial vaginosis. Obstet Gynecol 76:727–730, 1990.
24. Horowitz BJ, Mårdh P-A, Nagy E, Rank EL: Vaginal lactobacillosis. Am J Obstet Gynecol 170:857–861, 1994.
25. Holdeman LV, Cato GP, Moore WEL (eds): Anaerob Laboratory Manual, 4th ed. VPI Virginia Polytechnical Institute, Blacksburg, VA 1974.
26. Armitage P: Statistical Methods in Medical Research. Oxford: Blackwell Scientific Publications, pp 380–384, 1971.
27. Weström L: Diagnosis, aetiology and prognosis of acute salpingitis (thesis). Lund, Sweden: Studentlitteratur, pp 23–24, 1976.
28. Jonasson A, Larsson B, Bygdeman S, Forsum U: The influence of cervical dilation by laminaria tent and with hegar dilators on the intrauterine microflora and the rate of post-abortal pelvic inflammatory disease. Acta Obstet Gynecol Scand 68:405–410, 1989.
29. Levallois P, Riox JE: Prophylactic antibiotics for suction curettage abortion: Results of a clinical controlled trial. Am J Obstet Gynecol 158:100–105, 1988.
30. Holt JG, Krieg NR, Sneath PHA, Staley JT, Williams ST (eds): Genus *Lactobacillus*. In Bergery's Manual of Determinative Bacteriology, 9th ed. Baltimore: Williams & Wilkins, p 566, 1994.
